# Supplementary material for: Conditional chemoconnectomics (cCCTomics) as a strategy for efficient and conditional targeting of chemical transmission
Source: eLife. 2024 Apr 30;12:RP91927. doi: 10.7554/eLife.91927 (PMC11060718; doi:10.7554/eLife.91927)
Supplement: Figure 3—source data 3. [file elife-91927-fig3-data3.zip › Figure 3 source data3 part 1.zip!/Figure 3-Source data 3-part1/1175-9-CRISPResso_Report_rtnd62ko.zip!/CRISPResso_on_1175_9/1c.Alignment_barplot.pdf]

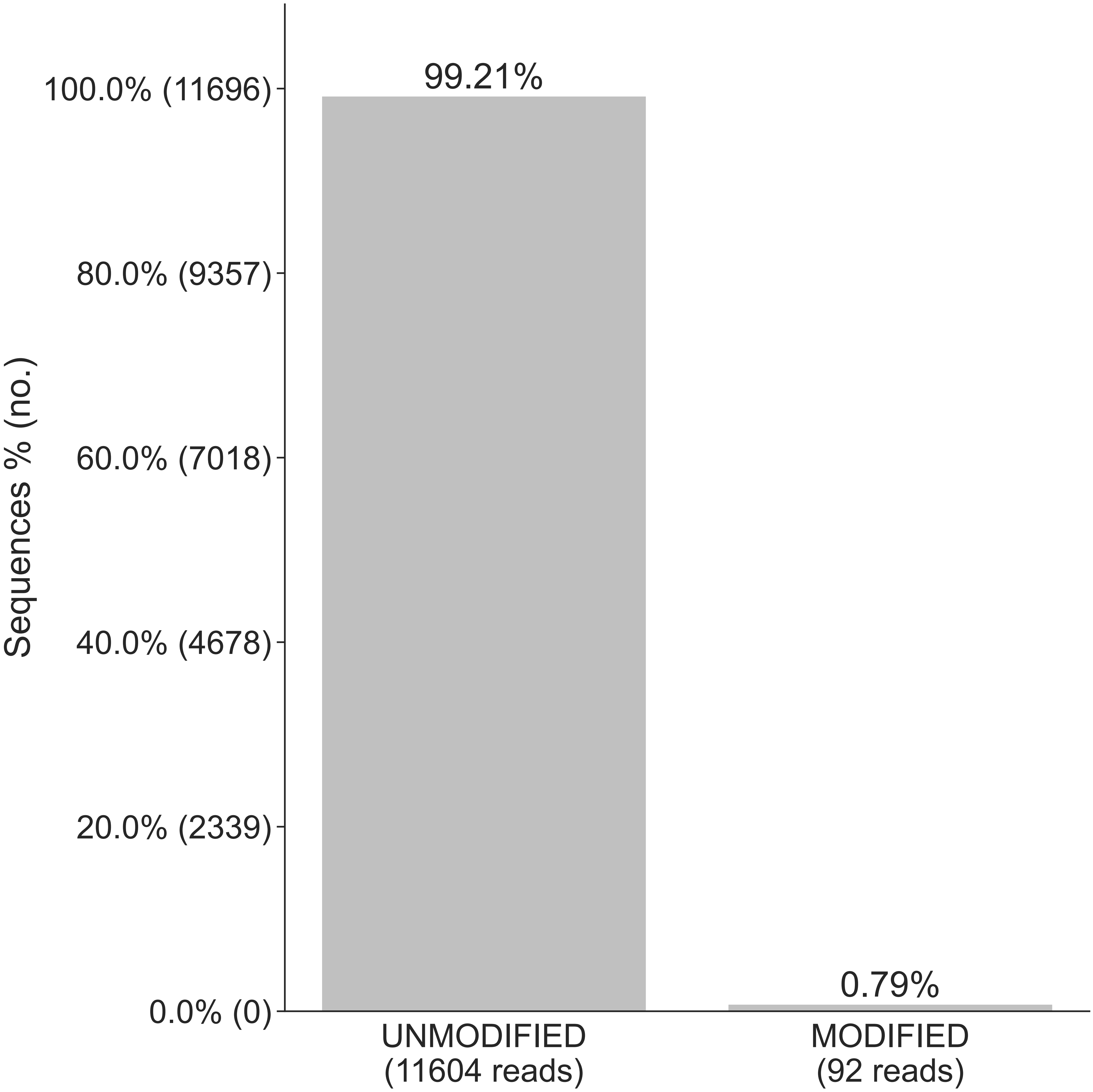

99.21%
0.79%
100.0% (11696)
80.0% (9357)
60.0% (7018)
40.0% (4678)
20.0% (2339)
0.0% (0)
Sequences % (no.)
UNMODIFIED
(11604 reads)
MODIFIED
(92 reads)
